# Announcement

**DOI:** 10.14252/foodsafetyfscj.D-26-00016

**Published:** 2026-06-26

**Authors:** Yasushi Yamazoe

**Affiliations:** Editor-in-Chief, *Food Safety*

Issue 2 of Volume 14 includes the peer-reviewed articles^1^^,^^2^^)^ presented at the 13th International Symposium “Approaches for risk analysis and food safety” organized by the Toxic Microorganisms Panel of the United States-Japan Cooperative Program on Development and Utilization of Natural Resources (UJNR), which was held at Seiryo Kaikan in Tokyo on September 17 and 18, 2024.

Dr. Matthew Wise*^1^ (Chairperson, US delegation) and Dr. Masashi Uema*^2^ (Chairperson, Japan delegation) kindly accepted to be guest editors for editing of these articles.

I wish to express my deep appreciation for their valuable effort in publication of the issues.
